# Response to college students’ mental health needs: a rapid review

**DOI:** 10.11606/s1518-8787.2021055003363

**Published:** 2021-12-20

**Authors:** Emiliana Maria Grando Gaiotto, Carla Andrea Trapé, Celia Maria Sivalli Campos, Elizabeth Fujimori, Fernanda Campos de Almeida Carrer, Lucia Yassuko Izumi Nichiata, Luciana Cordeiro, Maritsa Carla de Bortoli, Tatiana Yonekura, Tereza Setsuko Toma, Cassia Baldini Soares

**Affiliations:** I Universidade de São Paulo Escola de Enfermagem Departamento de Enfermagem em Saúde Coletiva São Paulo SP Brasil Universidade de São Paulo. Escola de Enfermagem. Departamento de Enfermagem em Saúde Coletiva. São Paulo, SP, Brasil; II Universidade de São Paulo Faculdade de Odontologia Departamento de Odontologia Social São Paulo SP Brasil Universidade de São Paulo. Faculdade de Odontologia. Departamento de Odontologia Social. São Paulo, SP, Brasil; III Universidade Federal de Pelotas Faculdade de Medicina Pelotas RS Brasil Universidade Federal de Pelotas, Faculdade de Medicina. Curso de Terapia Ocupacional. Pelotas, RS, Brasil; IV Secretaria de Estado da Saúde Instituto de Saúde São Paulo SP Brasil Secretaria de Estado da Saúde. Instituto de Saúde. São Paulo, SP, Brasil; V Hospital do Coração Laboratório de Implementação do Conhecimento em Saúde São Paulo SP Brasil Hospital do Coração. Laboratório de Implementação do Conhecimento em Saúde. São Paulo, SP, Brasil

**Keywords:** Students, Health Occupations, Student Health Services, Mental Health, Review

## Abstract

**OBJECTIVE:**

To present strategic options to support the adoption of mental health strengthening policies for university students in the field of health, to be implemented by university institutions.

**METHODS:**

Rapid review, without period delimitation, with searches carried out from May to June 2020, in 21 sources of bibliographic data, including gray literature. The following keywords were used: mental health, students and university. The selection process prioritized systematic reviews of mental health interventions for university students in health care courses, and also considered other types of review and relevant primary studies.

**RESULTS:**

Forty-five studies were included: 34 systematic reviews, an evidence synthesis, an overview*,* a scope review, three narrative reviews, three experience reports and two opinion articles. The evidence from these studies supported the development of four options: 1) to establish and support policies to strengthen the mental health of students in health care courses; 2) to integrate mental health care programs, expand their offer and facilitate access by students; 3) to promote educational programs and communication strategies related to contemporary psychic suffering and its confrontation, so that students can get to know the services and resources and identify strengthening practices; 4) to continuously monitor and assess the mental health needs of students in health care courses.

**CONCLUSIONS:**

The options are challenging and require universities to establish institutional commissions to implement a policy to strengthen the mental health of university students in the health area, with the ability to recognize the different health needs, including manifestations of psychic suffering ; to integrate the university’s internal actions with each other and with the services of the Unified Health System; to implement and monitor the actions that make up the mental health policy.

## INTRODUCTION

Mental health problems are a global concern and the complex phenomenon of suicide is considered a public health problem^1^because it is the second leading cause of death among young people aged 15 to 29 years^2^.

The object of this review is the mental suffering of university students, a group in which the phenomenon is associated with university sociability and family distancing^3,4^, as well as academic overload and an increasingly competitive environment generated by competition in the labor market^5^. For graduate students, the probability of suffering from depression and anxiety is six times greater than for the general population^9^.

A study conducted by the World Health Organization among university students from eight countries found that 35% of students had positive screening for at least one of the common mental disorders evaluated, reasons for suffering and impaired academic performance^10^.

An integrative review of the Brazilian literature found a variation from 34% to 49% in the prevalence of psychological distress among university students^11^. A survey conducted with 136,000 undergraduates, 14% of the total number of students from 53 Brazilian federal universities, found that 80% had emotional difficulties in the previous year, 58% related to anxiety, 45% to feelings of discouragement/lack of will, 32% to insomnia/sleep disorders, 23% to feeling helpless/hopeless, 21% to feelings of loneliness, 13% to eating problems and 11% to fear/panic. The results also identified 6% of responses related to the idea of death and 4% to suicidal thoughts, corresponding to almost 60,000 students who thought about death and 40,000 with suicidal ideation^12^.

In Brazil, studies that focus on health and education policies aimed at attention to mental suffering in university students mainly focus on students from courses in the health area^7,11^. The complexity of the health care object carries feelings that cause psychological distress^13^for students starting practical activities, due to insecurity and proximity to pain and death^3,11,14,15^. In nursing courses, exposure to stressful factors can occur during the initial adaptation period; throughout the course, due to insecurity and the complexity of care; in the end, due to the concern with entering the labor market and the demands of the profession^16^, and the traditional evaluation processes^17^. Undergraduates in the health area are more susceptible to psychological and emotional distress, due to the link to environments with high emotional demand, such as contact with pathological processes, like communicable diseases that generate fear of acquiring diseases, in addition to the fear of making mistakes and the feeling of impotence in the face of some diseases and death^3,14,15^.

It is noteworthy that mental problems starting in the university period can also affect professional life, which reinforces the importance and need for the development of institutional coping strategies, with the university environment being considered fertile for the conduct of actions that promote mental health^3, 4,14,18^.

Based on these considerations, this study aimed to present strategic options to support the adoption of institutional policies to strengthen the mental health of university students in the field of health, to be implemented by university institutions.

## METHODS

### Study Design

It is a rapid review, recognized as a type of study capable of providing high-quality evidence in a timely manner to support decision-making and the improvement of health policies, as per the guide of the World Health Organization (WHO) (2017 )^19^. The elaboration process is guided by the systematic review method, with adaptations, aiming to produce summaries of the best available evidence, in a timely manner, to meet specific demands^19^. This review was carried out in 90 days, a modality of the McMaster Health Forum Rapid Response program^a^, and developed in two stages. First, the problem was delimited through project team meetings and preliminary bibliographic surveys, which guided the stage of definition of search strategies and publications survey, to retrieve studies that presented or evaluated strengthening actions, programs and policies of mental health of university students, in order to compose a list of plausible interventions to be implemented by university institutions.

### Eligibility Criteria

Priority was given to studies of mental health interventions for university students, from systematic reviews (SR), with or without meta-analyses, overviews*,* evidence syntheses, and other types of reviews, published in English, Spanish and Portuguese. There was no restriction regarding the year of publication of the studies.

### Search and Selection of Studies

Searches were performed from May to June 2020, using the terms *students, university* and *mental health* in 21 data sources in the literature: PubMed, Health System Evidence, Social System Evidence, Epistemonikos, McMaster Plus, Health Evidence, Embase, ASSIA, Campbell, Cochrane, ERIC, JBI, CINAHL, Scopus, PsycInfo, LILACS, CAPES Theses and Dissertations Catalog, Sociological Abstract, OpenGrey, PEDro, Social Service Abstract. Search strategies were set out for each data source. Box 1 shows an example of the search strategy in PubMed.

**Box 1:** Search strategy in PubMed.

(((((((((((“Students, Medical”[Mesh] OR Medical Students OR Student, Medical OR Medical Student)) OR (“Students, Public Health”[Mesh] OR Health Student, Public OR Health Students, Public OR Public Health Student OR Student, Public Health OR Public Health Students)) OR (“Students, Nursing”[Mesh] OR Pupil Nurses OR Student, Nursing OR Nurses, Pupil OR Nurse, Pupil OR Pupil Nurse OR Nursing Student OR Nursing Students)) OR (((“Nutritionists”[Mesh] OR Nutritionist OR Dieticians OR Dietician OR Dietitians OR Dietitian)) AND (“Students”[Mesh] OR Student OR School Enrollment OR Enrollment, School OR Enrollments, School OR School Enrollments))) OR (((“Physical Therapists”[Mesh] OR Physical Therapist OR Therapist, Physical OR Therapists, Physical OR Physiotherapists OR Physiotherapist)) AND (“Students”[Mesh] OR Student OR School Enrollment OR Enrollment, School OR Enrollments, School OR School Enrollments))) OR (((“Occupational Therapists”[Mesh] OR Occupational Therapist OR Therapist, Occupational OR Therapists, Occupational)) AND (“Students”[Mesh] OR Student OR School Enrollment OR Enrollment, School OR Enrollments, School OR School Enrollments))) OR ((speech therapist) AND (“Students”[Mesh] OR Student OR School Enrollment OR Enrollment, School OR Enrollments, School OR School Enrollments)))) AND (((((“Universities”[Mesh] OR university)) OR (“Hospitals, University”[Mesh] OR university hospitals)) OR (“Academic Medical Centers”[Mesh] OR medical center, university OR university medical center OR medical centers, university OR university medical centers OR center, university medical OR center, academic medical OR centers, academic medical OR medical center, academic OR medical centers, academic OR centers, university medical OR academic medical center))) AND (((((((((((((“Mental Health”[Mesh] OR health, mental OR mental hygiene OR hygiene, mental)) OR (“Anxiety”[Mesh] OR hypervigilance OR nervousness OR social anxiety OR anxieties, social OR anxiety, social OR social anxieties)) OR (“Performance Anxiety”[Mesh] OR anxieties, performance OR anxiety, performance OR performance anxieties)) OR (“Anxiety Disorders”[Mesh] OR anxiety disorder OR disorder, anxiety OR disorders, anxiety OR neuroses, anxiety OR anxiety neuroses OR anxiety states, neurotic OR anxiety state, neurotic OR neurotic anxiety states OR neurotic anxiety states OR state, neurotic anxiety OR states, neurotic anxiety)) OR (“Phobia, Social”[Mesh] OR phobias, social OR social phobia OR social phobias OR social anxiety disorder OR anxiety disorder, social OR anxiety disorders, social OR disorder, social anxiety OR disorders, social anxiety OR social anxiety disorders)) OR (“Depression”[Mesh] OR depressions OR depressive symptoms OR depressive symptom OR symptom, depressive OR symptoms, depressive OR emotional depression OR depression, emotional OR depressions, emotional OR emotional depressions)) OR (“Depressive Disorder”[Mesh] OR depressive disorders OR disorder, depressive OR disorders, depressive OR neurosis, depressive OR depressive neuroses OR depressive neurosis OR neuroses, depressive OR depression, endogenous OR depressions, endogenous OR endogenous depression OR endogenous depressions OR depressive syndrome OR depressive syndromes OR syndrome, depressive OR syndromes, depressive OR depression, neurotic OR depressions, neurotic OR neurotic depression OR neurotic depressions OR melancholia OR melancholia OR unipolar depression OR depression, unipolar OR depressions, unipolar OR unipolar depressions)) OR (“Adjustment Disorders”[Mesh] OR reactive disorders OR disorder, reactive OR disorders, reactive OR reactive disorder OR adjustment disorder OR disorder, adjustment OR disorders, adjustment OR depression, reactive OR depressions, reactive OR reactive depression OR reactive depressions OR anniversary reaction OR anniversary reactions OR reaction, anniversary OR reactions, anniversary OR transient situational disturbance OR disturbance, transient situational OR disturbances, transient situational OR situational disturbance, transient OR situational disturbances, transient OR transient situational disturbances))) OR ((“Substance-Related Disorders”[Mesh] OR drug abuse OR abuse, drug OR drug dependence OR dependence, drug OR drug addiction OR addiction, drug OR substance use disorders OR disorder, substance use OR substance use disorder OR drug use disorders OR disorder, drug use OR drug use disorder OR organic mental disorders, substance-induced OR organic mental disorders, substance induced OR substance abuse OR abuse, substance OR abuses, substance OR substance abuse OR substance dependence OR dependence, substance OR substance addiction OR addiction, substance OR prescription drug abuse OR abuse, prescription drug OR drug abuse, prescription OR drug habituation OR habituation, drug))) OR ((((((“Stress, Psychological”[Mesh] OR psychological stress OR psychological stresses OR stresses, psychological OR life stress OR life stresses OR stress, life OR stresses, life OR stress, psychologic OR psychologic stress OR stressor, psychological OR psychological stressor OR psychological stressors OR stressors, psychological OR mental suffering OR suffering, mental OR suffering OR sufferings)) OR (“Occupational Stress”[Mesh] OR occupational stresses OR stress, occupational OR stresses, occupational OR job stress OR job stresses OR stress, job OR stresses, job OR work-related stress OR stress, work-related OR stresses, work-related OR work related stress OR work-related stresses OR workplace stress OR stress, workplace OR stresses, workplace OR workplace stresses OR workplace stress OR stress, workplace OR stresses, work place OR work place stresses OR professional stress OR professional stresses OR stress, professional OR stresses, professional OR job-related stress OR job related stress OR job-related stresses OR stress, job-related OR stresses, job-related)) OR (“Burnout, Psychological”[Mesh] OR psychological burnout OR burn-out syndrome OR burn out syndrome OR burnout OR burnout syndrome OR burn-out OR burn out OR psychological burn-out OR burn-out, psychological OR psychological burn out OR burnout, student OR school burnout OR student burnout OR burnout, school OR burnout, caregiver OR caregiver burnout OR caregiver exhaustion OR exhaustion, caregiver)) OR (“Burnout, Professional”[Mesh] OR professional burnout OR occupational burnout OR burnout, occupational OR career burnout OR burnout, career)) OR (“Workload”[Mesh] OR workloads OR work load OR work loads OR employee workload OR employee workloads OR workload, employee OR workloads, employee OR employee work load OR employee work load OR work load, employee OR work loads, employee OR staff workload OR staff workloads OR workload, staff OR workloads, staff OR staff work load OR staff work load OR work load, staff OR work loads, staff))) OR ((((“Suicide”[Mesh] OR suicides)) OR (“Suicide, Attempted”[Mesh] OR attempted suicide OR parasuicide OR parasuicides)) OR (“Suicide, Completed”[Mesh] OR completed suicides OR suicides, completed OR completed suicide)))))) AND (“Systematic Review” [Publication Type] OR Review, Systematic)

Subsequently, publications indicated by researchers or identified in supplementary searches were integrated to synthesize evidence not dealt with in the included reviews. The selection process showed that the contingent of publications on individual therapeutic interventions, of the cognitive-behavioral type, was very numerous (48), which would require extra time for data extraction. In this case, an additional filter, more rigorous and specific in the selection, was established, excluding reviews that did not provide the countries where the primary studies were carried out or the search date, and those that did not present a meta-analysis.

### Data Extraction and Assessment of the Methodological Quality of Included Studies

The extraction was performed in an Excel spreadsheet and included items such as author, year, study objective, intervention, results, limitations, proportion of studies from low- and middle- income countries, as classified by the World Bank^20^. The SRs were assessed for methodological quality using the AMSTAR^21^tool and classified as low (score 0 to 3), moderate (4 to 7) or high (8 to 11) quality. Non-systematic reviews and primary studies were also assessed for methodological quality using specific instruments: JBI Critical Appraisal Checklist for Text and Opinion Papers^22^; Critical Appraisal of a Case Study^23^; Scale for the Quality Assessment of Narrative Review Articles (SANRA)^24^; JBI Critical Appraisal Checklist for Systematic Reviews and Research Synthesis^25^; Criteria for Evaluation of Experience Report^26^and Evaluation of the Methodological Quality of Evidence Synthesis for Policy^27^. They were classified as low (up to 30%), moderate (30% to 60%) and high (60% to 100%) quality.

### Shortcuts Used

Six reviewers (CBS; EMGG; FCAC; MCB; LC; TST) performed the stages of study eligibility, data extraction and methodological quality assessment; as indicated in rapid reviews, the study did not need a pair of reviewers. Selection questions were resolved by consensus and extraction was verified by a seventh reviewer (TY).

## RESULTS

Search strategies retrieved 4,164 publications, 14 of which were duplicates and 4,047 were excluded in the steps of reading titles and abstracts. Of the 114 publications analyzed in full, 69 were excluded for not meeting the inclusion criteria, and finally 45 publications were included (Figure): 34 SR, one evidence synthesis, one overview, one scope review, three narrative reviews, three experience reports and two opinion articles.

**Figure:**
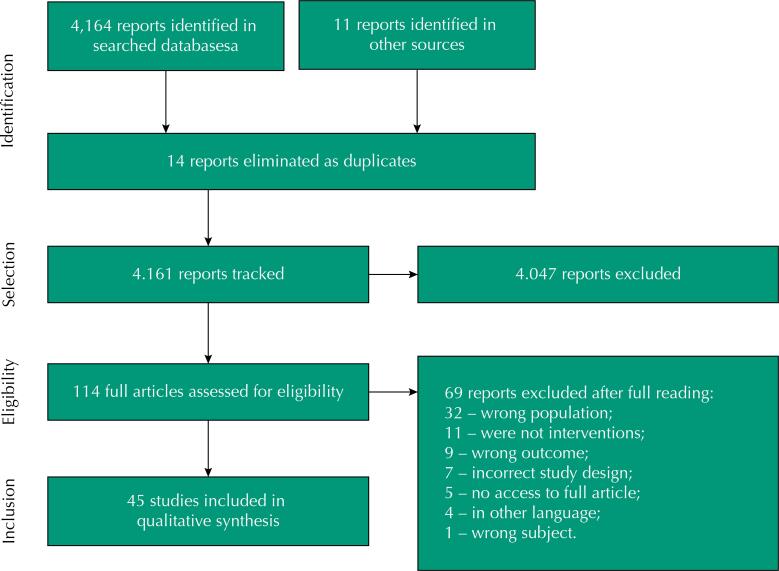
Flowchart of the selection of studies included in the rapid review. Note: ASSIA = 1; CAMPBELL = 337; CAPES = 57; CINAHL = 65; COCHRANE = 97; Embase = 487; Epistemonikos = 115; Eric = 38; Health Evidence = 38; Health Systems Evidence = 76; JBI = 58; LILACS = 46; McMaster Plus = 29; OpenGrey = 297; Pedro = 538; PsyInfo = 195; PubMed = 283; Scopus = 1,237; Social Service. .Abstract=14; Social Systems Evidence=123. Sociological Abstract=33.

Fonte: Adapted from Moher et al. (2009)^28^.

The studies included (Tables 1 and 2) provided evidence for the formulation of four strategic options to respond to students’ mental health needs. Each option presents a set of key messages, which constitute plausible courses of action for implementation (Box 2) of these options. The evidence behind these key messages is presented below.

**Table 1:** Main characteristics of the systematic reviews included.

Author (year)	Focus of systematic review	Number of studies included	Countries of included studies	AMSTAR article quality	Option
Amanvermez et al. (2020)^61^	Stress management programs.	54	Germany, Australia, Canada, Korea, Scotland and USA	7/11 moderate	2
Bakker et al. (2020)^45^	Interventions aimed at improving the mental health of students or novice nurses to prevent course and nursing work dropout.	21	Not reported	6/11 moderate	1
Bamber, Morpeth (2019)^51^	Effect of Mindfulness Meditation on Anxiety of College Students.	25	Australia, China, Korea, Spain, USA, UK and Thailand	7/11 moderate	2
Bolinski et al. (2020)^67^	Effectiveness of e-mental health interventions on academic performance of university students.	6	Australia, USA and UK	7/11 moderate	2
Chen et al. (2019)^54^	Effectiveness of non-pharmacological interventions in improving depressive symptoms and depression in nursing students.	13	Australia, China, Korea, USA, Iran, Thailand	8/11 high	2
Conley et al. (2017)^62^	Effectiveness of prevention programs designed for higher education students at risk for subsequent mental health difficulties.	60	Not reported	8/11 high	2
Conley et al. (2016)^70^	Technological interventions in mental health, aimed at higher education students without any mental health problem (universal prevention) or with mild to moderate subclinical problems (indicated prevention).	48	Australia, Canada, USA, Italy, Norway, Netherlands, UK and Romania	5/11 moderate	2
Conley, Durlak, Kirsch (2015)^59^	Skills training programs with supervised practice.	90	Belgium, Canada, Korea, Spain, USA, England, Japan, UK, Thailand and Turkey	7/11 moderate	2
Dawson et al. (2020)^55^	Effects of mindfulness-based interventions in a general population of college students.	51	Germany, Australia Canada, China, Singapore, Korea, Spain, USA, Netherlands, Japan, Malaysia, Norway, UK, Sweden and Thailand	9/11 high	2
El-Den et al. (2020)^47^	Evaluation of first aid training in mental health among college students.	12	Australia, USA and UK	3/11 low	1
Fernandez et al. (2016)^32^	Evaluation of structural and organizational strategies for promoting mental health in universities.	19	Not reported	6/11 moderate	1
Fu et al. (2020)^58^	Qualitative synthesis and effectiveness of psychological interventions in Chinese universities.	93	China	8/11 high	2
Ghilardi et al. (2017)^60^	Effectiveness of psychological interventions/individual or group counseling that meet the needs of university students.	16	Italy	5/11 moderate	2
González-Valero et al. (2019)^56^	Effect produced by cognitive-behavioral programs and meditation strategies on stress, anxiety and depression in university students.	34	Saudi Arabia, Australia, Belgium, China, South Korea, Spain, USA, Wales, England, Japan and Malaysia	7/11 moderate	2
Gulliver et al. (2015)^68^	Effectiveness of technology-based interventions for the use of tobacco and other drugs (except alcohol) in the university environment.	12	Germany, Canada, USA and Netherlands	8/11 high	2
Harrer et al. (2019)^71^	Effectiveness of virtual interventions for the mental health of university students.	48	Germany, Australia, Canada, Spain, USA, Finland, Ireland, Norway, UK, Romania and Sweden	8/11 high	2
Harrod et al. (2014)^31^	Primary suicide prevention interventions targeting college students and other post-high school courses to determine their effect on suicide and suicide-related outcomes.	8	Australia and USA	10/11 high	1 and 3
Heim et al. (2019)^37^	Interventions and methods applied to reduce mental health-related stigma among medical and nursing students.	9	Brazil, China, Malaysia, Nigeria, Somaliland and Turkey	7/11 moderate	1
Huang et al. (2018)^52^	Interventions for common mental health problems among college students.	51	Not reported	9/11 high	2
Johnson, Kalkbrenner (2017)^72^	Effect of the use of mHealth technology by university students to provide information related to well-being, identify what type of information is provided and students’ responsiveness.	13	USA and UK	4/11 moderate	3
Khadjesari et al. (2011)^69^	Effects of virtual interventions aimed at reducing alcohol consumption in adult populations.	24	Germany, USA, Netherlands, New Zealand and UK	5/11 moderate	2
Labrague et al. (2017)^41^	Identification of stress level and assessment of coping methods used by nursing students during the training course.	13	Australia, China, USA, Ghana, India, Iran, Ireland, Japan, Jordan and Taiwan	5/11 moderate	1
Leckey (2011)^38^	Effectiveness of creative activities on mental well-being in the context of mental health.	11	Not reported	4/11 moderate	1
Li et al. (2018)^57^	Effectiveness of interventions aimed at improving the mental health of nursing students.	12	Australia, China Korea, USA, UK, Sweden and Taiwan	8/11 high	2
Livingston et al. (2012)^34^	Evaluation of interventions aimed at reducing stigma related to substance use disorders.	13	Australia, Canada USA and UK	9/11 high	1
Mansfield et al. (2018)^39^	Effectiveness of participation in sport and dance on subjective well-being among healthy young people aged 15 to 24 years.	11	Not reported	9/11 high	1
Giralt-Palou, Prat-Vigué, Tort-Nasarre (2019)^35^	Nursing students’ perceptions of stigmatizing attitudes towards people who suffer from a mental health problem.	13	Not reported	6/11 moderate	1
Regehr, Glancy, Pitts (2013)^50^	Effectiveness of interventions aimed at reducing stress in college students.	32	Scotland, USA, Iran, Jordan, Switzerland and Tasmania	7/11 moderate	2
Reid, Carey (2015)^64^	Interventions with behavior change mechanisms, focusing on alcohol, for university students.	47	USA and others	6/11 moderate	2
Samson, Tanner- Smith (2015)^66^	Effectiveness of single-session interventions on alcohol consumption for college students.	73	Australia, Scandinavia, USA, New Zealand, UK and others	6/11 moderate	2
Shiralkar et al. (2013)^42^	How stress management programs were incorporated into the medical education curriculum for medical students and their impact on psychological distress.	13	Not reported	6/11 moderate	1
Winzer et al. (2018)^53^	Sustainability of the effects of interventions on the mental health of higher education students.	26	Australia, China, Scotland, Spain, USA, Hong Kong, Jordan and UK	9/11 high	2
Witt et al. (2019)^46^	Universal interventions for all students designed to address mental health problems, suicidal ideation, and behavior among medical students.	39	Germany, Saudi Arabia, Australia, Brazil, Canada, USA, India, Malaysia, Mexico, Norway, New Zealand and the United Kingdom	7/11 moderate	1 and 2
Yamaguchi et al. (2013)^36^	Reduction of stigma related to mental health problems.	35	Germany, Australia, USA, Japan, New Zealand, UK, Russia, Taiwan and Turkey	7/11 moderate	1

**Table 2:** Main characteristics of non-systematic reviews and primary studies included.

Author/Year	Study design	Study countries	Study focus	Study quality	Option
Dietz et al. (2020)^63^	Umbrella review	Germany, China, USA, Italy, Portugal, Poland and UK	Interventions to promote modifiable factors that influence the health of university students.	10/10 high	2
Maito et al. (2019)^49^	Case study	Brazil	Proposal of institutional forms and normative-dogmatic parameters for fighting violence, emphasizing the legal responsibility of higher education institutions.	8/10 high	1 and 3
Meredith et al. (2020)^40^	Scope review	USA, Japan and Sweden	Definition of a time measure of activities in nature that cause positive impacts on the mental health and well-being of university students.	8/10 high	1
Okanlawon (2021)^48^	Narrative review	Nigeria	Overview of homophobic and heterosexist experiences and challenges that LGBT students face in Nigerian schools and universities, such as rejection, bullying and victimization.	12/9 high	1
Reavley, Jorm (2010)^65^	Narrative review	Not reported	Prevention and early intervention in mental health problems (anxiety, depression and alcohol abuse among higher education students).	11/12 high	2
Tamboly, Gauvin (2013)^33^	Evidence synthesis	Canada	Mental health needs of McMaster University students and coping options.	19/21 high	1,3 and 4
Cordeiro et al. (2016)^43^	Experience report	Brazil	Subject program of “Psychoactive Drugs: Education and Harm Reduction”, as well as the projects proposed by students.	7/7 high	1
Bleicher (2018)^29^	Experience report	Brazil	Mental health actions developed at the Federal University of São Carlos and its project to align with the perspective of psychosocial care.	7/7 high	1, 2 and 4
Metivier (2020)^30^	Opinion article	USA	Components of reforms needed for anti-racist university education.	6/6 high	1
Leonello et al. (2018)^74^	Experience report	Brazil	Pedagogical follow-up activity and support to students, carried out by the Coordinating Committee of the Bachelor’s Degree in Nursing at the USP School of Nursing.	6/7 high	4
Cromlish (2020)^44^	Opinion article	USA and Finland	Comparison of US and Finnish universities in relation to mental health in higher education.	6/6 high	1 and 4

**Box 2:** Options and key messages.

Options for policies	Key message
**Option 1:** Establish and support a policy to strengthen student mental health in the health area, capable of sensitizing and responding to the identification of mental health needs.	1. Organize a mental health committee within the courses in the health area, with the objective of discussing the main problems that affect students and supporting them in deciding on the best way to face the challenges they are facing; 2. Develop activities to reduce the stigma related to mental health, as well as to promote cultural improvement, well-being and sociability, which can contribute to a change in mentality and strengthen students in the search for improvement in mental health; 3. Propose curricular changes in courses in the health area of the University in order to provide critical elements for students to understand the relationship between mental health and society, detect problems, seek help and support colleagues who are experiencing situations of psychic suffering; 4. Integrate programs and activities to combat racism and prejudice against LGBTI, quota students, and other forms of discrimination against students, as well as programs and activities to support student retention.
**Option 2:** Integrate and expand the provision of mental health care programs and provide access to them for health students.	5. Formally integrate mental health care services available at the university through a collaborative network of health services offered on and off campus, integrated with SUS services, developing a flow of comprehensive care for university students; 6. Expand health promotion activities and non-pharmacological interventions in mental health services offered by the university; 7. Adopt an assessment program, using virtual technologies, as an application to assess the mental health of university students for referral to appropriate health services.
**Option 3:** Promote educational programs and communication strategies for students in the health area, regarding psychic suffering in contemporary times and ways of coping, to ensure that students know about services and resources, identify welcoming practices and can access them.	8. Promote communication and make available, in an accessible way, information from all health services and support groups for students focused on mental health, through a cell phone application and links on the university’s official websites; 9. Encourage discussions about current youth problems and stimulate critical debate about racism, prejudice and all forms of discrimination that affect students’ mental health.
**Option 4:** Continuously monitor and assess the mental health needs of students in health courses.	10. Implement a mental health monitoring program in courses in the health area, for the planning and implementation of actions; 11. Create a support network from a mentoring program to monitor students.

**Key message 1.** Based on the policy of the Federal University of São Carlos (UFSCar), which integrates the various actors in mental health, establishes a working group to define a common agenda and organizes a front to face suicide^29^. To compose this policy, principles and actions to combat racism must be considered, adopting reforms of commitment to rights, justice, dignity, respect, participation and intersectoriality, which involves the public recognition of institutional responsibility in combating racism, and adoption of mechanisms to identify and fix problems and provide support for coping^30^. Multicomponent suicide prevention policies involved restricting the means to commit suicide and mandatory health assessment for those with suicidal behavior^31,32^.

**Key message 2.** Based on evidence on the availability of resources and training to faculty to create a stigma-free university environment, and on encouraging the development of student bodies to support those with mental health problems^33^; interventions to reduce stigma through training students on the theme^34-37^and activities that promote cultural and social development and that favor well-being, such as theater, dance, nature walks^38-40^and movies, for improvement patterns of rest and sleep^41^.

**Key message 3.** Based on: introduction of stress management programs into the medical curriculum, changes in duration, curriculum type, and pass/fail grading system^42^; interprofessional discipline on contemporary youth problems, with emancipatory potential for university sociability^43^; curriculum changes implemented in early periods of courses to increase social skills and resources to address personal or academic problems^44^; mandatory course on mental health in modern society and a course in mind-body medicine, taught to first-year medical students, as well as structural changes in the curriculum^32;^ group stress management, training in relaxation skills and cognitive-behavioral techniques in the Nursing course to prevent course dropout^45^; structural, systemic and cultural changes that can impact medical education^46^. Although training programs in early mental health care are considered effective to improve knowledge in the area, few are the curricula that integrate them, being found in courses in the health area in only three countries^47^.

**Key message 4.** Supported by interventions to improve access to minority mental health services, such as crisis services information hotline, presentations of experts and family members and individuals with mental health problems^33^. The organization of conferences on inclusion, equality and diversity in university education provided an opportunity to discuss homophobia for academics, students, LGBT activists and other Nigerian groups^47^. Institutional guidelines for situations of violence and discrimination based on gender and sexual orientation, and protection measures so that victims of violence are not harmed in their training, such as comprehensive care for victims, investigation and rapid responses to reported cases^48^.

**Key message 5.** Based on the concept of integration of services and resources offered by the university to those of the Brazilian Unified Health System (SUS), as integrated strategies can enhance the supply and access to services, preventing the university from taking responsibility alone for the care of students^29^.

**Key message 6.** Supported by evidence on individual non-pharmacological treatments for stress, anxiety and other signs and symptoms of psychic suffering. Analysis of mindfulness intervention and other behavioral therapies showed satisfactory results^46,50-56^. For depression, psychological therapies were highlighted as the most effective^57,58^. Mindfulness interventions and stress management programs were effective. Compared to pharmacological treatment, non-pharmacological interventions had moderate beneficial effects on depressive symptoms in nursing students. Short-term interventions moderately relieved depressive symptoms and depression^54^. Psychoeducational interventions produced significant effects in reducing symptoms of anxiety, stress, psychological distress, among others^59^. Interventions with music, physical exercise, yoga, tai chi, among other activities, were effective in preventing common mental health problems, with medium-term programs having better effects than short-term ones^52^. Counseling and mindfulness interventions contributed to stress management and reduction^60-62^. Brief interventions with individual focus of mindfulness were shown to be limited in reducing levels of anxiety, depression and stress in medical students with suicidal ideation; most of the evaluated interventions were offered during the pre-clinical years, and there is evidence that the problems become more expressive during the period of incursion into clinical practice^46^. Analyses of interventions to reduce alcohol consumption, such as face-to-face and internet programs, have shown the need for further research to identify more promising approaches^63^. Individual therapies aimed at changing behavior showed an effect in reducing alcohol consumption^64,65^. Satisfactory results were observed from the brief, single-session intervention for high alcohol consumption, but future research should examine what would be the effective duration of this intervention^66^. Regarding the improvement of eating habits, face-to-face interventions, media approaches and nutrition labeling were positive. Physical activity promotion interventions should carefully consider personalized interventions. In the case of sleep, cognitive behavioral therapy showed greater effects compared to hygiene interventions^63^.

**Key message 7.** Based on e-health type interventions*.* A small effect on academic performance, depression and anxiety was reported in the analysis of interventions such as: web platform, with optional use of a mobile application; program with personalized feedback; opportunities for personal training integrated into the university’s online course platform; intervention with thematic modules such as goal setting, personal strengths and career plan; integration of knowledge about oneself with meaningful goals; intervention based on acceptance and commitment therapy; computerized expressive writing intervention to report academic fears^67^. Multidirectional intervention, with feedback through a computer program, showed a positive result on the intention to smoke cigarettes, but not marijuana. Brief web-based or computer-based personalized feedback programs were not effective in reducing or preventing marijuana use^68^. Virtual interventions such as feedback to assess current levels of alcohol consumption and interactive games have shown potential to help reduce alcohol consumption^69^. Indicated interventions (with pre-existing problems) were advantageous over universal interventions (without pre-existing problems) for outcomes such as depression, anxiety, stress, social and emotional skills^70^. Internet interventions to improve mental health, well-being, and social and academic functioning showed small positive effects for depression, anxiety and stress symptoms, and moderate positive effects for eating disorder symptoms and social and academic functioning^71^.

**Key message 8.** Considered strategies such as: articulation between mental health services inside and outside the university; presentation of these services to students; access to resource information materials available in print and online; mental health campaigns; sending e mail to students; and teacher training to address mental health issues, including referrals to appropriate services^33^ Evidence was also integrated about the use of the app to inform about the services offered in health and reception units at the university, and about well-being. The information was considered convenient and reliable by the students, being effective in reducing anxiety and depression symptoms; risk identification for mental disorders; reduction of alcohol and tobacco consumption and cessation of tobacco consumption^71^. Educational program and gatekeeper training with the objective that colleagues, teachers and employees recognize and respond to the warning signs of emotional crises and suicide risk were considered effective, with an increase in short-term knowledge and self-efficacy in suicide prevention^31^.

**Key message 9.** Formulated based on an intervention at the University of Mexico, which created reception and support services for students, and sought to understand the situation of the university community, offer courses on gender for teachers and advocate for the training of university surveillance workers^49^.

**Key message 10.** Based on the monitoring model of the University of Dentistry of São Paulo (FOUSP), conducted through longitudinal research of a cohort composed of freshmen, emphasizing the burnout syndrome. Another strategy involving application of research with feedback and follow-up of undergraduate and graduate students is used to improve mental health and culture at the university, since professors and other workers monitor the results (available anonymously). The feedback involves time management, better study and metacognitive skills, as well as preparation for the specific difficulties of the programs^44^. UFSCar develops longitudinal research following the university community and created an extension program involving the academic departments of courses in the health area, the University Hospital, among other entities, having as its axis the institutional diagnosis, the profile of mental health in the university community and articulation with the municipality’s psychosocial care network^29^.

**Key message 11.** Based on experience with the development of tutoring programs in two units of the University of São Paulo (USP): FOUSP, which provides resources such as a website*,* tutor guide with basic guidelines for teachers, annual training for tutors with mental health professionals and semiannual follow-up reports, with 20 professors, a psychologist and two social workers supporting undergraduate students^73^; the School of Nursing, which develops an academic tutoring project, with four professors from the Bachelor’s Degree Commission, with one being a reference for each undergraduate year, for monitoring academic difficulties, listening to suffering and strain related to mental health and referrals^74^. McMaster University student housing workers and members of student unions collaborate with first-year undergraduates who struggle to transition to university; in addition, professors are encouraged to offer students, in the disciplines, availability to discuss mental health problems, such as stress and anxiety; other groups also provide support to students^33^.

## DISCUSSION

The options formulated from the literature to respond to the mental health needs of university students are challenging and require universities to establish institutional commissions capable of implementing a policy in the area.

The first option summons managers of healthcare courses and universities to action and decision-making, showing the need to establish institutional policies, in contrast to specific actions and isolated initiatives. This decision requires the constitution of a working group that: prepares a plan for implementation, with clear and objectively delineated purposes and goals; provide the development and monitoring of the results of projects and implemented actions; and offer permanent support to students and managers, for the effectiveness and continuity of the process. These actions must not lose sight of the confrontation of the stigma intrinsic to the theme of mental suffering, nor the perspective of dealing with the theme of mental health across the curriculum, in order to enable the student to develop critical analysis and the understanding of the roots of problems faced. The studies point to the need to include teachers and other workers in the discussion process, improving the debate and favoring proposals for joint confrontation, with institutional commitment to sensitive issues that cause suffering, such as racism, prejudices against LGBTI and quota holders, and other forms of discrimination^48,75^. This first option requires deep structural, paradigm and practice changes from the institutions, and its implementation in the medium/long term demands dedication of time and effort from those involved.

As a second option, in the short term, universities and courses in the health area can make an effort to identify existing actions and available services, in order to integrate them and formally offer the university community information and access to this care network. Institutional protagonism is essential, organizing programs and actions linked to the university’s internal care network and SUS (Brazilian Unified Health System), to the detriment of isolated actions. The need to expand the offer of these services is included, pointing out two paths: expansion of therapeutic activities so that they are not restricted to pharmacological interventions, and inclusion of information and communication technologies to assess suffering, then guiding and directing students to appropriate services. Telehealth is a useful tool to increase access, and mental health is a pioneer in the use of these technologies^67,71,72^. The university, which often researches these innovations, has a duty to incorporate them into the care routine of its community^16,17^.

Option 3 focuses on the need for institutional development of educational strategies that illuminate contemporary mental problems, with emphasis on those arising from prejudice and attitudes of discrimination, and the ways in which they are faced. The implementation of educational strategies and debates with the entire academic community should focus on issues related to forms of discrimination and their relationship with psychic suffering. This debate can bring up demands and strengthen the intra-institutional support network^31,33^.

Option 4 shows that coping with psychic suffering requires permanent monitoring of mental health actions, as well as their inclusion in the institutional agenda, with the creation of support networks for students and the involvement of the entire academic community. In this option, tutoring programs and monitoring of cases of mental suffering among students are identified, within the units, which demand coping and welcoming strategies, joint reflection on the identified problems and possible referrals, follow-up and monitoring of the processes^28,43,73^. The institution can create integrated work mechanisms with health services, family and professors, to facilitate the student’s therapeutic process, given that the university environment is excellent for the implementation and monitoring of actions to promote mental health^4,19^.

Mental health needs are shaped by the forms of work and life inherent to the class inclusion of students and their families^78^. A university policy to strengthen university students in the health area, which takes into account the strategic options shown in the literature, must recognize the social differences and the different manifestations of students’ psychological suffering, in the implementation of collective monitoring mechanisms.

In health courses, the conditions for admission and permanence, as well as the occurrence of mental health problems, are unequal. An analysis of the occurrence of depressive symptoms among students from different courses shows a higher prevalence of these symptoms in nursing courses, followed by dentistry and medicine courses^79^. These courses are attended by graduates from heterogeneous groups from the point of view of class insertion, considering social indicators such as the level of education of father and mother and type of institution (public/private) where the student attended high school^80^.

Recognizing that the university environment can be, in part, the cause of mental suffering is a fundamental step in transforming the university into a healthier environment.

To cope with psychological suffering, UFSCar constituted a commission that proposed a mental health policy^b^ that provides for the integration between SUS services and the services and resources offered by the university, in order to develop actions aimed at: improving mental health; prevention of injuries; the provision of care to consequences of these possible injuries, such as suicide attempts; and the reduction of harm caused by the problematic use of psychoactive substances. It also provides for actions to collect, analyze and manage data to generate indicators and monitor the phenomena of psychological distress and evaluate the actions taken. It also indicates the establishment of mechanisms to understand the relationship between the teaching/learning processes and psychic suffering, as well as the development of the UFSCar Code of Ethics, and protocols for preventive actions and for the care of situations of violence^81^.

The options presented here, for coping with the psychic suffering of students, require the involvement of the entire academic community, with a marked commitment from the faculty, which, however, is under intense pressure and psychological burden^82^. In the Brazilian context, this is compounded by scientific productivity demands to professors linked to graduate programs, which generate suffering and illness^83^.

The subjective precariousness felt by the university professor, who is permanently concerned with responding to the high productivity demands, translates into a feeling of isolation and abandonment^84^. This precariousness operates less objectively as compared to that which affects workers from outsourced companies and temporary professors, as they are subjected to informal work and the loss of fundamental, social, labor and social security rights. Therefore, they see their security concretely shaken.

Graduate students have also been the object of studies related to stress in mental health^9,85,86^, as a consequence of pressure. There are pressures caused by the obligation to fulfill academic demands; by the difficulty in maintaining a balance between academic and personal life; by uncertainties about the future^85^; and by the need to achieve the academic productivity goals required by research development agencies^86^.

Therefore, the implementation of responses to mental health needs within universities finds the context of pressure for productivism, which imposes on professors, students and the entire set of workers the achievement of institutional goals based on quantitative international standards of academic excellence. The deepening and amplification of neoliberalism in higher education needs to be made explicit and recognized as a process that responds to the aggressive global capitalist expansion, shaped by the market logic^87,88^. In the Brazilian case, the quality of knowledge production is questioned in the face of the current competition for excellence^89^.

It is urgent that universities make commitments to the public cause, genuinely democratic, for and with society. Since neoliberalism is ideologically pedagogical, in the sense of teaching consent and reproducing domination, the answer, also of a pedagogical nature, must radically oppose it, constituting a transformative pedagogy, whose purpose is freedom and emancipation^90^.

This study has some limitations: it did not survey evidence of harm reduction, an approach increasingly adopted by institutions, in the area of harmful drug use among university students; it included some non-systematic reviews and primary studies, due to the need to address the collective and organizational dimensions and other related objects that were not addressed in systematic reviews.
