# Supplementary material for: Response to college students’ mental health needs: a rapid review
Source: Rev Saude Publica. 2021 Dec 20;55:114. doi: 10.11606/s1518-8787.2021055003363 (PMC8687654; doi:10.11606/s1518-8787.2021055003363)
Supplement: Translation

# Translation: Resposta a necessidades em saúde mental de estudantes universitários: uma revisão rápida

**Authors:** Emiliana Maria Grando Gaiotto, Carla Andrea Trapé, Celia Maria Sivalli Campos, Elizabeth Fujimori, Fernanda Campos de Almeida Carrer, Lucia Yassuko Izumi Nichiata, Luciana Cordeiro, Maritsa Carla de Bortoli, Tatiana Yonekura, Setsuko Tereza Toma, Cassia Baldini Soares

**Affiliations:** I Brasil Universidade de São Paulo. Escola de Enfermagem. Departamento de Enfermagem em Saúde Coletiva. São Paulo, SP, Brasil; II Brasil Universidade de São Paulo. Faculdade de Odontologia. Departamento de Odontologia Social. São Paulo, SP, Brasil; III Brasil Universidade Federal de Pelotas, Faculdade de Medicina. Curso de Terapia Ocupacional. Pelotas, RS, Brasil; IV Brasil Secretaria de Estado da Saúde. Instituto de Saúde. São Paulo, SP, Brasil; V Brasil Hospital do Coração. Laboratório de Implementação do Conhecimento em Saúde. São Paulo, SP, Brasil

**Keywords:** Estudantes de Ciências da Saúde, Serviços de Saúde para Estudantes, Saúde Mental, Revisão

## Abstract

**OBJETIVO:**

Apresentar opções estratégicas para apoiar a adoção de políticas de fortalecimento da saúde mental de universitários da área da saúde, a serem implementadas por instituições universitárias.

**MÉTODOS:**

Revisão rápida, sem delimitação de período, com buscas realizadas de maio a junho de 2020, em 21 fontes de dados bibliográficos, incluindo literatura cinzenta. Utilizaram-se as palavras-chave: saúde mental, estudantes e universidade. O processo de seleção priorizou revisões sistemáticas sobre intervenções em saúde mental para estudantes universitários em cursos da área da saúde, e considerou, também, outros tipos de revisão e estudos primários relevantes.

**RESULTADOS:**

Foram incluídos 45 estudos: 34 revisões sistemáticas, uma síntese de evidências, um *overview,* uma revisão de escopo, três revisões narrativas, três relatos de experiência e dois artigos de opinião. As evidências desses estudos apoiaram a elaboração de quatro opções: 1) estabelecer e apoiar políticas de fortalecimento da saúde mental de estudantes dos cursos da área da saúde; 2) integrar programas de atenção à saúde mental, ampliar sua oferta e facilitar seu acesso pelos estudantes; 3) promover programas educacionais e estratégias de comunicação relacionadas ao sofrimento psíquico contemporâneo e ao seu enfrentamento, para que os estudantes conheçam os serviços e recursos e identifiquem práticas de fortalecimento; 4) monitorar e avaliar continuamente as necessidades em saúde mental dos estudantes dos cursos da área da saúde.

**CONCLUSÕES:**

As opções são desafiadoras e exigem que as universidades estabeleçam comissões institucionais para implementar uma política de fortalecimento da saúde mental dos estudantes universitários da área da saúde, com capacidade de reconhecer as diversas necessidades em saúde, incluindo as manifestações de sofrimento psíquico; integrar ações internas da universidade entre si e aos serviços do Sistema Único de Saúde; implementar e monitorar as ações que compõem a política de saúde mental.

## INTRODUÇÃO

Problemas de saúde mental constituem preocupação mundial e o complexo fenômeno do suicídio é considerado problema de saúde pública^1^ por ser a segunda maior causa de mortes entre jovens de 15 a 29 anos^2^.

O objeto desta revisão é o sofrimento mental de universitários, grupo em que o fenômeno se associa à sociabilidade universitária e ao distanciamento familiar^3,4^, bem como à sobrecarga letiva e à ambiência cada vez mais competitiva gerada pela concorrência no mercado de trabalho^5^. Para os pós-graduandos, a probabilidade de sofrer depressão e ansiedade é seis vezes maior do que para a população em geral^9^.

Estudo conduzido pela Organização Mundial da Saúde, entre universitários de oito países, constatou que 35% dos estudantes apresentavam triagem positiva para ao menos um dos transtornos mentais comuns avaliados, motivos de sofrimento e prejuízo ao desempenho acadêmico^10^.

Revisão integrativa da literatura brasileira constatou variação de 34% a 49% na prevalência de sofrimento psíquico entre universitários^11^. Pesquisa realizada com 136 mil graduandos, 14% do total de alunos de 53 universidades federais brasileiras, verificou que 80% tiveram dificuldades emocionais no ano anterior, sendo 58% relacionadas a quadros de ansiedade, 45% a sentimento de desânimo/falta de vontade, 32% a insônia/alterações de sono, 23% a sentimento de desamparo/desesperança, 21% a sentimentos de solidão, 13% a problemas alimentares e 11% a medo/pânico. Os resultados identificaram ainda 6% de respostas relacionadas à ideia de morte e 4% a pensamento suicida, correspondendo a quase 60 mil discentes que pensaram em morte e 40 mil com ideação suicida^12^.

No Brasil, os estudos que tomam como objeto políticas de saúde e de educação voltadas à atenção ao sofrimento mental em universitários, focalizam principalmente estudantes de cursos da área da saúde^7,11^. A complexidade do objeto de cuidado em saúde carrega sentimentos que causam sofrimento psíquico^13^ de estudantes em início de atividades práticas, pela insegurança e pela proximidade com dor e morte^3,11,14,15^. Nos cursos de enfermagem, a exposição a fatores estressantes pode ocorrer no período de adaptação inicial; ao longo do curso, por insegurança e pela complexidade do cuidado; no final, pela preocupação com a inserção no mercado de trabalho e as exigências da profissão^16^, e pelos processos avaliativos tradicionais^17^. Os graduandos da área da saúde são mais suscetíveis ao desgaste psicológico e emocional, pelo vínculo a ambientes com elevada demanda emocional, como o contato com processos patológicos, como doenças transmissíveis que geram medo de adquirir doenças, além do receio de cometer erros e sentimento de impotência frente a algumas doenças e morte^3,14,15^.

Ressalta-se que problemas mentais iniciados no período universitário podem também afetar a vida profissional, o que reforça a importância e a necessidade da elaboração de estratégias de enfrentamento institucional, sendo o ambiente universitário considerado fecundo para a condução de ações promotoras de saúde mental^3,4,14,18^.

A partir dessas considerações, este estudo teve como objetivo apresentar opções estratégicas para apoiar a adoção de políticas institucionais de fortalecimento da saúde mental de universitários da área da saúde, a serem implementadas por instituições universitárias.

## MÉTODOS

### Delineamento do Estudo

Trata-se de revisão rápida, reconhecida como um tipo de estudo capaz de fornecer evidências de alta qualidade e de forma oportuna para apoiar a tomada de decisão e o aperfeiçoamento das políticas de saúde, conforme guia da Organização Mundial da Saúde (OMS) (2017)^19^. O processo de elaboração é orientado pelo método da revisão sistemática, com adaptações, visando produzir sínteses das melhores evidências disponíveis, em tempo hábil, para atender demandas específicas^19^. A presente revisão foi realizada em 90 dias, modalidade do programa de Respostas Rápidas do McMaster Health Forum^a^, e elaborada em duas etapas. Primeiramente, delimitou-se o problema por meio de reuniões da equipe do projeto e levantamentos bibliográficos preliminares, o que orientou a etapa de definição das estratégias de busca e levantamento das publicações, para recuperar estudos que apresentassem ou avaliassem ações, programas e políticas de fortalecimento da saúde mental de estudantes universitários, de forma a compor um rol de intervenções plausíveis de serem implementadas por instituições universitárias.

### Critérios de Elegibilidade

Incluíram-se prioritariamente estudos de intervenções em saúde mental para estudantes universitários, provenientes de revisões sistemáticas (RS), com ou sem metanálises, *overviews,* sínteses de evidências, e outros tipos de revisão, publicadas em inglês, espanhol e português. Não houve restrição quanto ao ano de publicação dos estudos.

### Busca e Seleção dos Estudos

As buscas foram realizadas de maio a junho de 2020, com os termos *“students”, “university”* e *“mental health”*, em 21 fontes de dados da literatura: PubMed, Health System Evidence, Social System Evidence, Epistemonikos, McMaster Plus, Health Evidence, Embase, ASSIA, Campbell, Cochrane, ERIC, JBI, CINAHL, Scopus, PsycInfo, Lilacs, Catálogo de Teses e Dissertações da CAPES, Sociological Abstract, OpenGrey, PEDro, Social Service Abstract. As estratégias de busca foram equacionadas para cada fonte de dados. O Quadro 1 mostra exemplo da estratégia de busca no PubMed.

**Quadro 1:** Estratégia de busca no PubMed.

(((((((((((“Students, Medical”[Mesh] OR Medical Students OR Student, Medical OR Medical Student)) OR (“Students, Public Health”[Mesh] OR Health Student, Public OR Health Students, Public OR Public Health Student OR Student, Public Health OR Public Health Students)) OR (“Students, Nursing”[Mesh] OR Pupil Nurses OR Student, Nursing OR Nurses, Pupil OR Nurse, Pupil OR Pupil Nurse OR Nursing Student OR Nursing Students)) OR (((“Nutritionists”[Mesh] OR Nutritionist OR Dieticians OR Dietician OR Dietitians OR Dietitian)) AND (“Students”[Mesh] OR Student OR School Enrollment OR Enrollment, School OR Enrollments, School OR School Enrollments))) OR (((“Physical Therapists”[Mesh] OR Physical Therapist OR Therapist, Physical OR Therapists, Physical OR Physiotherapists OR Physiotherapist)) AND (“Students”[Mesh] OR Student OR School Enrollment OR Enrollment, School OR Enrollments, School OR School Enrollments))) OR (((“Occupational Therapists”[Mesh] OR Occupational Therapist OR Therapist, Occupational OR Therapists, Occupational)) AND (“Students”[Mesh] OR Student OR School Enrollment OR Enrollment, School OR Enrollments, School OR School Enrollments))) OR ((speech therapist) AND (“Students”[Mesh] OR Student OR School Enrollment OR Enrollment, School OR Enrollments, School OR School Enrollments)))) AND (((((“Universities”[Mesh] OR university)) OR (“Hospitals, University”[Mesh] OR university hospitals)) OR (“Academic Medical Centers”[Mesh] OR medical center, university OR university medical center OR medical centers, university OR university medical centers OR center, university medical OR center, academic medical OR centers, academic medical OR medical center, academic OR medical centers, academic OR centers, university medical OR academic medical center))) AND (((((((((((((“Mental Health”[Mesh] OR health, mental OR mental hygiene OR hygiene, mental)) OR (“Anxiety”[Mesh] OR hypervigilance OR nervousness OR social anxiety OR anxieties, social OR anxiety, social OR social anxieties)) OR (“Performance Anxiety”[Mesh] OR anxieties, performance OR anxiety, performance OR performance anxieties)) OR (“Anxiety Disorders”[Mesh] OR anxiety disorder OR disorder, anxiety OR disorders, anxiety OR neuroses, anxiety OR anxiety neuroses OR anxiety states, neurotic OR anxiety state, neurotic OR neurotic anxiety states OR neurotic anxiety states OR state, neurotic anxiety OR states, neurotic anxiety)) OR (“Phobia, Social”[Mesh] OR phobias, social OR social phobia OR social phobias OR social anxiety disorder OR anxiety disorder, social OR anxiety disorders, social OR disorder, social anxiety OR disorders, social anxiety OR social anxiety disorders)) OR (“Depression”[Mesh] OR depressions OR depressive symptoms OR depressive symptom OR symptom, depressive OR symptoms, depressive OR emotional depression OR depression, emotional OR depressions, emotional OR emotional depressions)) OR (“Depressive Disorder”[Mesh] OR depressive disorders OR disorder, depressive OR disorders, depressive OR neurosis, depressive OR depressive neuroses OR depressive neurosis OR neuroses, depressive OR depression, endogenous OR depressions, endogenous OR endogenous depression OR endogenous depressions OR depressive syndrome OR depressive syndromes OR syndrome, depressive OR syndromes, depressive OR depression, neurotic OR depressions, neurotic OR neurotic depression OR neurotic depressions OR melancholia OR melancholia OR unipolar depression OR depression, unipolar OR depressions, unipolar OR unipolar depressions)) OR (“Adjustment Disorders”[Mesh] OR reactive disorders OR disorder, reactive OR disorders, reactive OR reactive disorder OR adjustment disorder OR disorder, adjustment OR disorders, adjustment OR depression, reactive OR depressions, reactive OR reactive depression OR reactive depressions OR anniversary reaction OR anniversary reactions OR reaction, anniversary OR reactions, anniversary OR transient situational disturbance OR disturbance, transient situational OR disturbances, transient situational OR situational disturbance, transient OR situational disturbances, transient OR transient situational disturbances))) OR ((“Substance-Related Disorders”[Mesh] OR drug abuse OR abuse, drug OR drug dependence OR dependence, drug OR drug addiction OR addiction, drug OR substance use disorders OR disorder, substance use OR substance use disorder OR drug use disorders OR disorder, drug use OR drug use disorder OR organic mental disorders, substance-induced OR organic mental disorders, substance induced OR substance abuse OR abuse, substance OR abuses, substance OR substance abuse OR substance dependence OR dependence, substance OR substance addiction OR addiction, substance OR prescription drug abuse OR abuse, prescription drug OR drug abuse, prescription OR drug habituation OR habituation, drug))) OR ((((((“Stress, Psychological”[Mesh] OR psychological stress OR psychological stresses OR stresses, psychological OR life stress OR life stresses OR stress, life OR stresses, life OR stress, psychologic OR psychologic stress OR stressor, psychological OR psychological stressor OR psychological stressors OR stressors, psychological OR mental suffering OR suffering, mental OR suffering OR sufferings)) OR (“Occupational Stress”[Mesh] OR occupational stresses OR stress, occupational OR stresses, occupational OR job stress OR job stresses OR stress, job OR stresses, job OR work-related stress OR stress, work-related OR stresses, work-related OR work related stress OR work-related stresses OR workplace stress OR stress, workplace OR stresses, workplace OR workplace stresses OR workplace stress OR stress, workplace OR stresses, work place OR work place stresses OR professional stress OR professional stresses OR stress, professional OR stresses, professional OR job-related stress OR job related stress OR job-related stresses OR stress, job-related OR stresses, job-related)) OR (“Burnout, Psychological”[Mesh] OR psychological burnout OR burn-out syndrome OR burn out syndrome OR burnout OR burnout syndrome OR burn-out OR burn out OR psychological burn-out OR burn-out, psychological OR psychological burn out OR burnout, student OR school burnout OR student burnout OR burnout, school OR burnout, caregiver OR caregiver burnout OR caregiver exhaustion OR exhaustion, caregiver)) OR (“Burnout, Professional”[Mesh] OR professional burnout OR occupational burnout OR burnout, occupational OR career burnout OR burnout, career)) OR (“Workload”[Mesh] OR workloads OR work load OR work loads OR employee workload OR employee workloads OR workload, employee OR workloads, employee OR employee work load OR employee work load OR work load, employee OR work loads, employee OR staff workload OR staff workloads OR workload, staff OR workloads, staff OR staff work load OR staff work load OR work load, staff OR work loads, staff))) OR ((((“Suicide”[Mesh] OR suicides)) OR (“Suicide, Attempted”[Mesh] OR attempted suicide OR parasuicide OR parasuicides)) OR (“Suicide, Completed”[Mesh] OR completed suicides OR suicides, completed OR completed suicide)))))) AND (“Systematic Review” [Publication Type] OR Review, Systematic)

Integrou-se, posteriormente, publicações indicadas por pesquisadores ou identificadas em buscas suplementares para sintetizar evidências não tratadas nas revisões incluídas. O processo de seleção mostrou que o contingente de publicações sobre intervenções terapêuticas individuais, do tipo cognitivo-comportamentais, era muito numeroso (48), o que demandaria tempo extra para a extração dos dados. Nesse caso, estabeleceu-se um filtro adicional, mais rigoroso e específico na seleção, excluindo as revisões que não informaram os países onde os estudos primários foram realizados, a data da busca, e que não apresentaram metanálise.

### Extração dos Dados e Avaliação da Qualidade Metodológica dos Estudos Incluídos

A extração foi realizada em planilha Excel e incluiu itens como autor, ano, objetivo do estudo, intervenção, resultados, limitações, proporção de estudos de países de baixa e média renda, conforme classificação do Banco Mundial^20^. As RS foram avaliadas quanto à qualidade metodológica por meio da ferramenta AMSTAR^21^e classificadas em qualidade baixa (escore 0 a 3), moderada (4 a 7) ou alta (8 a 11). As revisões não sistemáticas e os estudos primários também foram avaliados quanto à qualidade metodológica por meio de instrumentos específicos: JBI Critical Appraisal Checklist for Text and Opinion Papers^22^; Critical Appraisal of a Case Study^23^; Scale for the Quality Assessment of Narrative Review Articles (SANRA)^24^; JBI Critical Appraisal Checklist for Systematic Reviews and Research Synthesis^25^; Critérios para Avaliação de Relato de Experiência^26^ e Avaliação da Qualidade Metodológica de Síntese de Evidências para Política^27^. Foram classificadas em qualidade baixa (até 30%), moderada (30% a 60%) e alta (60% a 100%).

### Atalhos Utilizados

Seis revisoras (CBS; EMGG; FCAC; MCB; LC; TST) realizaram as etapas de elegibilidade dos estudos, extração de dados e avaliação da qualidade metodológica; como indicado em revisões rápidas, o estudo prescindiu de dupla de revisores. As dúvidas de seleção foram resolvidas por consenso e a extração foi verificada por uma sétima revisora (TY).

## RESULTADOS

As estratégias de busca recuperaram 4.164 publicações, dentre as quais 14 eram duplicadas e 4.047 foram excluídas nas etapas de leitura de títulos e resumos. Das 114 publicações analisadas na íntegra, 69 foram excluídas por não atenderem aos critérios de inclusão, sendo finalmente incluídas 45 publicações (Figura): 34 RS, uma síntese de evidências, um *overview*, uma revisão de escopo, três revisões narrativas, três relatos de experiência e dois artigos de opinião.

**Figura:** Fluxograma da etapa de seleção de estudos incluídos na revisão rápida. Nota: ASSIA = 1; CAMPBELL = 337; CAPES = 57; CINAHL = 65; COCHRANE = 97; Embase = 487; Epistemonikos = 115; Eric = 38; Health Evidence = 38; Health Systems Evidence = 76; JBI = 58; Lilacs = 46; McMaster Plus = 29; OpenGrey = 297; Pedro = 538; PsyInfo = 195; PubMed = 283; Scopus = 1.237; Social Service Abstract = 14; Social Systems Evidence = 123; Sociological Abstract = 33.

Fonte: Adaptado de Moher et al. (2009)^28^.

Os estudos incluídos (Tabelas 1 e 2) ofereceram evidências para a formulação de quatro opções estratégicas para responder às necessidades em saúde mental dos estudantes. Cada opção apresenta um conjunto de mensagens-chave, que constituem linhas de ação plausíveis para implementação (Quadro 2) dessas opções. A seguir se apresentam as evidências que fundamentam essas mensagens-chave.

**Tabela 1:** Principais características das revisões sistemáticas incluídas.

Autor (ano)	Foco da revisão sistemática	Nº de estudos incluídos	Países dos estudos incluídos	AMSTAR qualidade do artigo	Opção
Amanvermez et al. (2020)^61^	Programas de gestão de estresse.	54	Alemanha, Austrália, Canadá, Coreia, Escócia e EUA	7/11 moderada	2
Bakker et al. (2020)^45^	Intervenções destinadas a melhorar a saúde mental de estudantes ou enfermeiros novatos para prevenir o abandono do curso ou o trabalho em enfermagem.	21	Não reportado	6/11 moderada	1
Bamber, Morpeth (2019)^51^	Efeito da meditação de atenção plena sobre a ansiedade de estudantes universitários.	25	Austrália, China, Coreia, Espanha, EUA, Reino Unido e Tailândia	7/11 moderada	2
Bolinski et al. (2020)^67^	Eficácia de intervenções e-mental health (saúde mental digital) no desempenho acadêmico de estudantes universitários.	6	Austrália, EUA e Reino Unido	7/11 moderada	2
Chen et al. (2019)^54^	Efetividade de intervenções não farmacológicas na melhora de sintomas depressivos e depressão de estudantes de enfermagem.	13	Austrália, China, Coreia, EUA, Irã, Tailândia	8/11 alta	2
Conley et al. (2017)^62^	Eficácia dos programas de prevenção indicados para estudantes do ensino superior em risco de subsequentes dificuldades de saúde mental.	60	Não reportado	8/11 alta	2
Conley et al. (2016)^70^	Intervenções tecnológicas na saúde mental, voltadas para estudantes do ensino superior sem qualquer problema de saúde mental (prevenção universal) ou com problemas subclínicos leves a moderados (prevenção indicada).	48	Austrália, Canadá, EUA, Itália, Noruega, Países Baixos, Reino Unido e Romênia	5/11 moderada	2
Conley, Durlak, Kirsch (2015)^59^	Programas de treinamento de habilidades com prática supervisionada.	90	Bélgica, Canadá, Coreia, Espanha, EUA, Inglaterra, Japão, Reino Unido, Tailândia e Turquia	7/11 moderada	2
Dawson et al. (2020)^55^	Efeitos de intervenções baseadas em atenção plena em uma população geral de estudantes universitários.	51	Alemanha, Austrália Canadá, China, Cingapura, Coreia, Espanha, EUA, Holanda, Japão, Malásia, Noruega, Reino Unido, Suécia e Tailândia	9/11 alta	2
El-Den et al. (2020)^47^	Avaliação do treinamento Primeiros Socorros em Saúde Mental entre estudantes universitários.	12	Austrália, EUA e Reino Unido	3/11 baixa	1
Fernandez et al. (2016)^32^	Avaliação de estratégias estruturais e organizacionais para a promoção da saúde mental em universidades.	19	Não reportado	6/11 moderada	1
Fu et al. (2020)^58^	Síntese qualitativa e efetividade de intervenções psicológicas em universidades chinesas.	93	China	8/11 alta	2
Ghilardi et al. (2017)^60^	Eficácia das intervenções psicológicas/ aconselhamentos individuais ou grupais que atendem às necessidades de estudantes universitários.	16	Itália	5/11 moderada	2
González-Valero et al. (2019)^56^	Efeito produzido por programas cognitivo- comportamentais e estratégias de meditação sobre o estresse, ansiedade e depressão em estudantes universitários.	34	Arábia Saudita, Austrália, Bélgica, China, Coreia do Sul, Espanha, EUA, Gales, Inglaterra, Japão e Malásia	7/11 moderada	2
Gulliver et al. (2015)^68^	Eficácia das intervenções baseadas em tecnologia para o uso de tabaco e outras drogas (exceto álcool) no ambiente universitário.	12	Alemanha, Canadá, EUA e Holanda	8/11 alta	2
Harrer et al. (2019)^71^	Eficácia de intervenções virtuais para a saúde mental de estudantes universitários.	48	Alemanha, Austrália, Canadá, Espanha, EUA, Finlândia, Irlanda, Noruega, Reino Unido, Romênia e Suécia	8/11 alta	2
Harrod et al. (2014)^31^	Intervenções primárias de prevenção do suicídio direcionadas a alunos universitários e outros cursos pós ensino médio para determinar seu efeito no suicídio e nos resultados relacionados ao suicídio.	8	Austrália e EUA	10/11 alta	1 e 3
Heim et al. (2019)^37^	Intervenções e métodos aplicados para redução do estigma relacionado à saúde mental entre estudantes de medicina e enfermagem.	9	Brasil, China, Malásia, Nigéria, Somalilândia e Turquia	7/11 moderada	1
Huang et al. (2018)^52^	Intervenções para problemas comuns de saúde mental entre estudantes universitários.	51	Não reportado	9/11 alta	2
Johnson, Kalkbrenner (2017)^72^	Efeito do uso da tecnologia mHealth, por estudantes universitários, para fornecer informações relacionadas ao bem-estar, identificar que tipo de informação é fornecida e a capacidade de resposta dos estudantes.	13	EUA e Reino Unido	4/11 moderada	3
Khadjesari et al. (2011)^69^	Efeitos das intervenções virtuais destinadas a reduzir o consumo de álcool em populações adultas.	24	Alemanha, EUA, Holanda, Nova Zelândia e Reino Unido	5/11 moderada	2
Labrague et al. (2017)^41^	Identificação do nível de estresse e avaliação dos métodos de enfrentamento utilizados por estudantes de enfermagem durante o curso de formação.	13	Austrália, China, EUA, Gana, Índia, Irã, Irlanda, Japão, Jordânia e Taiwan	5/11 moderada	1
Leckey (2011)^38^	Eficácia das atividades criativas no bem-estar mental no contexto da saúde mental.	11	Não reportado	4/11 moderada	1
Li et al. (2018)^57^	Efetividade das intervenções destinadas a melhorar a saúde mental dos estudantes de enfermagem.	12	Austrália, China Coreia, EUA, Reino Unido, Suécia e Taiwan	8/11 alta	2
Livingston et al. (2012)^34^	Avaliação das intervenções destinadas a reduzir o estigma relacionado aos transtornos por uso de substâncias.	13	Austrália, Canadá EUA e Reino Unido	9/11 alta	1
Mansfield et al. (2018)^39^	Eficácia da participação no esporte e na dança no bem-estar subjetivo entre jovens saudáveis de 15 anos a 24 anos.	11	Não reportado	9/11 alta	1
Giralt-Palou, Prat-Vigué, Tort-Nasarre (2019)^35^	Percepções dos alunos de enfermagem sobre atitudes estigmatizantes em relação às pessoas que sofrem de um problema de saúde mental.	13	Não reportado	6/11 moderada	1
Regehr, Glancy, Pitts (2013)^50^	Eficácia de intervenções destinadas a reduzir o estresse em estudantes universitários.	32	Escócia, EUA, Irã, Jordânia, Suíça e Tasmânia	7/11 moderada	2
Reid, Carey (2015)^64^	Intervenções com mecanismos de mudança de comportamento, com foco no álcool, para estudantes universitários.	47	EUA e Outros	6/11 moderada	2
Samson, Tanner- Smith (2015)^66^	Efetividade de intervenções de sessões únicas sobre consumo de bebidas alcoólicas para estudantes universitários.	73	Austrália, Escandinávia, EUA, Nova Zelândia, Reino Unido e Outros	6/11 moderada	2
Shiralkar et al. (2013)^42^	Como os programas de gerenciamento de estresse foram incorporados ao currículo de educação médica para estudantes de medicina e seu impacto no sofrimento psicológico.	13	Não reportado	6/11 moderada	1
Winzer et al. (2018)^53^	Sustentabilidade dos efeitos de intervenções na saúde mental de estudantes de nível superior.	26	Austrália, China, Escócia, Espanha, EUA, Hong Kong, Jordânia e Reino Unido	9/11 alta	2
Witt et al. (2019)^46^	Intervenções universais a todos os alunos destinadas a abordar problemas de saúde mental, ideação suicida e comportamento entre estudantes de medicina.	39	Alemanha, Arábia Saudita, Austrália, Brasil, Canadá, EUA, Índia, Malásia, México, Noruega, Nova Zelândia e Reino Unido	7/11 moderada	1 e 2
Yamaguchi et al. (2013)^36^	Redução do estigma relacionado aos problemas de saúde mental.	35	Alemanha, Austrália, EUA, Japão, Nova Zelândia, Reino Unido, Rússia, Taiwan e Turquia	8/11 alta	1

**Tabela 2:** Principais características das revisões não sistemáticas e dos estudos primários incluídos.

Autor/Ano	Desenho do estudo	Países dos estudos	Foco do estudo	Qualidade do estudo	Opção
Dietz et al. (2020)^63^	Revisão guarda-chuva	Alemanha, China, EUA, Itália, Portugal, Polônia e Reino Unido	Intervenções para promover fatores modificáveis que influenciam a saúde em estudantes universitários.	10/10 alta	2
Maito et al. (2019)^49^	Estudo de caso	Brasil	Proposta de formas institucionais e parâmetros normativo- dogmáticos para o enfrentamento da violência, enfatizando a responsabilidade jurídica das instituição de ensino superior.	8/10 alta	1 e 3
Meredith et al. (2020^)40^	Revisão de escopo	EUA, Japão e Suécia	Definição de medida de tempo de atividades em meio à natureza que causam impactos positivos na saúde mental e no bem-estar de estudantes universitários.	8/10 alta	1
Okanlawon (2021)^48^	Revisão narrativa	Nigéria	Quadro geral das experiências e desafios homofóbicos e heterossexistas que os alunos LGBT enfrentam nas escolas e universidades nigerianas, como rejeição, bullying e vitimização.	9/12 alta	1
Reavley, Jorm (2010)^65^	Revisão narrativa	Não reportado	Prevenção e intervenção precoce em problemas de saúde mental (ansiedade, depressão e uso indevido de álcool entre estudantes de ensino superior).	11/12 alta	2
Tamboly, Gauvin (2013)^33^	Síntese de evidências	Canadá	Necessidades em saúde mental dos estudantes da Universidade McMaster e as opções para enfrentamento.	19/21 alta	1,3 e 4
Cordeiro et al. (2016)^43^	Relato de experiência	Brasil	Programa da disciplina “Drogas Psicoativas: Educação e Redução de Danos”, bem como os projetos propostos pelos estudantes.	7/7 alta	1
Bleicher (2018)^29^	Relato de experiência	Brasil	Ações em saúde mental desenvolvidas na Universidade Federal de São Carlos e seu projeto de alinhamento com a perspectiva da atenção psicossocial.	7/7 alta	1, 2 e 4
Metivier (2020)^30^	Artigo de opinião	EUA	Componentes de reformas necessárias à formação universitária antirracista.	6/6 alta	1
Leonello et al. (2018)^74^	Relato de experiência	Brasil	Atividade de acompanhamento pedagógico e apoio aos estudantes, realizada pela Comissão Coordenadora do curso de bacharelado em Enfermagem da Escola de Enfermagem da USP.	6/7 alta	4
Cromlish (2020)^44^	Artigo de opinião	EUA e Finlândia	Comparação de universidades dos EUA e da Finlândia em relação à saúde mental no ensino superior.	6/6 alta	1 e 4

**Quadro 2:** Opções e mensagens-chave.

Opções para políticas	Mensagem chave
**Opção 1:** Estabelecer e dar suporte a uma política de fortalecimento da saúde mental de estudante da área saúde, capaz de sensibilizar para a identificação de necessidades em saúde mental, bem como dar respostas a elas.	1. Construir um comitê de saúde mental no âmbito dos cursos da área de saúde, com o objetivo de discutir os principais problemas que afetam os estudantes e apoiá-los na decisão sobre a melhor maneira de enfrentar os desafios que estão vivendo; 2. Desenvolver atividades para diminuição do estigma relacionado à saúde mental, bem como para promover o aperfeiçoamento cultural, o bem-estar e a sociabilidade, que podem contribuir para mudança de mentalidade e fortalecimento dos estudantes na busca por melhoria da saúde mental; 3. Propor mudanças curriculares nos cursos da área da saúde na Universidade no sentido de proporcionar elementos críticos aos estudantes para que compreendam a relação entre saúde mental e sociedade, detectem problemas, busquem ajuda e apoiem colegas que estão passando por situações de sofrimento psíquico; 4. Integrar programas e atividades de combate ao racismo e preconceitos contra LGBTI, cotistas, e outras formas de discriminação dos estudantes, bem como programas e atividades de apoio à permanência dos estudantes.
**Opção 2:** Integrar e ampliar a oferta de programas de atenção em saúde mental e proporcionar acesso a eles, para estudantes da área da saúde.	5. Integrar formalmente os serviços de atenção em saúde mental disponibilizados na universidade por meio de rede colaborativa de serviços de saúde oferecidos dentro e fora do campus, integrada aos serviços do SUS, elaborando fluxo de cuidados integrais aos estudantes universitários; 6. Ampliar as atividades de promoção da saúde e intervenções não farmacológicas nos serviços de saúde mental oferecidos pela universidade; 7. Adotar programa de avaliação, com uso de tecnologias virtuais, como aplicativo para avaliação da saúde mental de estudantes universitários para encaminhamento aos serviços de saúde apropriados.
**Opção 3:** Promover programas educativos e estratégias comunicacionais para os estudantes da área da saúde, quanto ao sofrimento psíquico na contemporaneidade e às formas de enfrentamento, para assegurar que os estudantes conheçam os serviços e recursos, identifiquem práticas acolhedoras e possam acessá-los.	8. Promover a comunicação e disponibilizar, de maneira acessível, informações oriundas de todos os serviços de saúde e grupos de apoio a estudantes voltados à saúde mental, por meio de aplicativo de celular e *links* nos *sites* oficiais da universidade; 9. Incentivar discussões sobre os problemas atuais da juventude e estimular o debate crítico sobre racismo, preconceitos e todas as formas de discriminação que afetam a saúde mental dos estudantes.
**Opção 4:** Monitorar e avaliar continuamente as necessidades em saúde mental de estudantes dos cursos da saúde.	10. Implementar programa de monitoramento em saúde mental nos cursos da área da saúde, para o planejamento e implementação de ações; 11. Criar uma rede de apoio a partir de programa de tutoria para acompanhamento de estudantes.

**Mensagem-chave 1.** Baseia-se em política da Universidade Federal de São Carlos (UFSCar), que integra os diversos atores em saúde mental, estabelece um grupo de trabalho para definição de agenda comum e organiza uma frente para o enfrentamento do suicídio^29^. Para compor essa política, deve-se considerar princípios e ações de enfrentamento do racismo, adotando reformas de compromisso com direitos, justiça, dignidade, respeito, participação e intersetorialidade, o que envolve o reconhecimento público da responsabilidade institucional no combate ao racismo, e a adoção de mecanismos para identificar e corrigir problemas e oferecer suporte ao enfrentamento^30^. Políticas de multicomponentes de prevenção ao suicídio envolveram restrição de meios para praticar o suicídio e avaliação de saúde obrigatória para os que apresentam comportamento suicida^31,32^.

**Mensagem-chave 2.** Fundamenta-se em evidências sobre a oferta de recursos, no treinamento do corpo docente para criar ambiente universitário livre de estigma, e no estímulo ao desenvolvimento de entidades estudantis de apoio aos que apresentam problemas de saúde mental^33^; intervenções para reduzir estigma por meio da capacitação de estudantes no tema^34-37^ e de atividades que propiciem desenvolvimento cultural e social, e que favoreçam o bem-estar, como teatro, dança, caminhadas na natureza^38-40^ e filmes, para a melhoria dos padrões de descanso e sono^41^.

**Mensagem-chave 3.** Baseia-se em: introdução de programas de gerenciamento de estresse no currículo médico, mudanças na duração, no tipo de currículo e no sistema de notas de aprovação ou reprovação^42^; disciplina interprofissional sobre os problemas da juventude na contemporaneidade, com potencialidades emancipatórias da sociabilidade universitária^43^; mudanças curriculares implementadas em períodos iniciais dos cursos para aumentar as habilidades sociais e recursos para abordar os problemas pessoais ou acadêmicos^44^; curso obrigatório sobre saúde mental na sociedade moderna e curso de medicina mente-corpo, ministrado aos alunos do primeiro ano de Medicina, bem como mudanças estruturais no currículo^32^, gerenciamento de estresse em grupo, treinamento de habilidades de relaxamento e técnicas cognitivo-comportamentais no curso de Enfermagem para prevenir o abandono do curso^45^; mudanças de cunho estrutural, sistêmico e cultural que podem impactar a formação médica^46^. Ainda que programas de formação em primeiros cuidados de saúde mental sejam considerados efetivos para melhorar o conhecimento na área, poucos são os currículos que os integram, sendo encontrados em cursos na área da saúde em apenas três países^47^.

**Mensagem-chave 4.** Apoia-se em intervenções para melhorar o acesso a serviços de saúde mental de minorias, como linha direta de informações sobre serviços para crises, apresentações de especialistas e de familiares e indivíduos com problemas de saúde mental^33^. A organização de conferências sobre inclusão, igualdade e diversidade na educação universitária proporcionou oportunidade de discutir a homofobia para acadêmicos, estudantes, ativistas LGBT e outros grupos nigerianos^48^. Diretrizes institucionais para situações de violência e discriminação de gênero e orientação sexual, e medidas de proteção para que vítimas de violência não tenham prejuízos na sua formação, como atendimento integral aos vitimados, investigação e respostas rápidas aos casos notificados^49^.

**Mensagem-chave 5.** Fundamenta-se em conceito de integração dos serviços e recursos oferecidos pela universidade aos do Sistema Único de Saúde (SUS), pois as estratégias integradas podem potencializar a oferta e o acesso a serviços, evitando que a universidade se responsabilize isoladamente pelo cuidado dos estudantes^29^.

**Mensagem-chave 6.** Apoia-se nas evidências sobre tratamentos individuais não farmacológicos para estresse, ansiedade e outros sinais e sintomas de sofrimento psíquico. As análises de intervenção de atenção plena (*mindfulness*) e outras terapias comportamentais mostraram resultados satisfatórios^46,50-56^. Para depressão foram destacadas como mais efetivas as terapias psicológicas^57,58^. Intervenções de atenção plena e programas de gerenciamento de estresse foram eficazes. Comparadas ao tratamento farmacológico, as intervenções não farmacológicas tiveram efeitos benéficos moderados sobre os sintomas depressivos em estudantes de Enfermagem. Intervenções de curto prazo aliviaram moderadamente os sintomas depressivos e a depressão^54^. Intervenções psicoeducacionais produziram efeitos significativos na redução de sintomas de ansiedade, estresse, sofrimento psicológico, entre outros^59^. Intervenções com música, exercícios físicos, yoga, tai chi, entre outras atividades, foram eficazes para prevenir problemas comuns de saúde mental, sendo que os programas de duração média apresentaram melhores efeitos que os de curta duração^52^. Intervenções de aconselhamento e atenção plena colaboraram para gerenciamento e diminuição do estresse^60-62^. Intervenções breves e com foco individual de atenção plena mostraram-se limitadas para a redução dos níveis de ansiedade, depressão e estresse em estudantes de Medicina com ideação suicida; a maior parte das intervenções avaliadas foi oferecida durante os anos pré-clínicos, sendo que há evidências de que os problemas tornam-se mais expressivos no período de incursão na prática clínica^46^. Análises das intervenções para a redução do consumo de álcool, como programas face a face e via internet, mostraram a necessidade de futuras pesquisas para identificar abordagens mais promissoras^63^. Terapias individuais voltadas à mudança de comportamento mostraram efeito na redução do consumo de álcool^64,65^. Foram observados resultados satisfatórios da intervenção breve, de sessão única, para o consumo elevado de álcool, mas pesquisas futuras devem examinar qual seria a duração eficaz dessa intervenção^66^. Com relação à melhoria dos hábitos alimentares, intervenções presenciais, abordagens de mídia e rotulagem nutricional mostraram-se positivas. As intervenções de promoção de atividade física devem considerar com cautela a personalização. No caso do sono, a terapia cognitiva comportamental mostrou efeitos maiores em comparação com as intervenções de higiene^63^.

**Mensagem-chave 7.** Baseia-se em intervenções do tipo *e-health.* Foi relatado efeito pequeno sobre o desempenho acadêmico, depressão e ansiedade na análise de intervenções como: plataforma web, com uso opcional de aplicativo de celular; programa com *feedback* personalizado; oportunidades para capacitação pessoal integrada à plataforma de cursos online da universidade; intervenção com módulos temáticos como definição de metas, pontos fortes pessoais e plano de carreira; integração do conhecimento sobre si mesmo com objetivos significativos; intervenção baseada em terapia de aceitação e compromisso; intervenção de escrita expressiva computadorizada, para relato de receios acadêmicos^67^. Intervenção multidirecionada, com *feedback* por meio de programa computacional, mostrou resultado positivo sobre a intenção de fumar cigarros, mas não maconha. Programas breves de *feedback* personalizado baseados na web ou no computador não foram eficazes na redução ou prevenção do uso de maconha^68^. Intervenções virtuais como *feedback* para avaliar os níveis atuais de consumo de álcool e jogos interativos mostraram potencial para ajudar a reduzir o consumo de álcool^69^. Intervenções indicadas (com problemas pré-existentes) foram vantajosas em relação a intervenções universais (sem problemas pré-existentes) para desfechos como depressão, ansiedade, estresse, habilidades sociais e emocionais^70^. Intervenções via internet para a melhoria da saúde mental, do bem-estar e do funcionamento social e acadêmico mostraram efeitos positivos pequenos para a depressão, ansiedade e sintomas de estresse, e moderados para os sintomas de transtorno alimentar e para o funcionamento social e acadêmico^71^.

**Mensagem-chave 8.** Considerou-se estratégias como: articulação entre serviços de saúde mental dentro e fora da universidade; apresentação desses serviços aos estudantes; acesso a materiais informativos sobre recursos disponíveis em forma impressa e on-line; campanhas sobre saúde mental; envio de e-mail aos estudantes; e treinamento de professores para abordar questões relativas à saúde mental, inclusive encaminhamentos a serviços apropriados^33^. Integrou-se, ainda, evidência sobre o uso de aplicativo para informar sobre os serviços oferecidos em unidades de saúde e acolhimento na universidade, e sobre o bem-estar. As informações foram consideradas convenientes e confiáveis pelos estudantes, sendo efetivas para redução de sintomas de ansiedade e depressão; identificação de risco para transtornos mentais; redução do consumo de álcool e tabaco e cessação do consumo de tabaco^71^. Programa educacional e treinamento de *gatekeepers* com objetivo de que colegas, professores e funcionários reconheçam e respondam aos sinais de alerta de crises emocionais e risco de suicídio foram considerados efetivos, com aumento do conhecimento de curto prazo e autoeficácia na prevenção de suicídio^31^.

**Mensagem-chave 9.** Formulada com base em intervenção na Universidade do México, que criou serviços de acolhimento e apoio para estudantes, e buscou conhecer a situação da comunidade universitária, oferecer cursos sobre gênero para docentes e defender a capacitação dos trabalhadores da vigilância universitária^49^.

**Mensagem-chave 10.** Apoia-se no modelo de monitoramento da Faculdade de Odontologia da Universidade de São Paulo (FOUSP), realizado por meio de pesquisa longitudinal, de coorte formado por ingressantes, com ênfase na síndrome de *Burnout*. Outra estratégia envolvendo aplicação de pesquisa com *feedback* e acompanhamento dos estudantes de graduação e pós-graduação é usada para melhorar a saúde mental e a cultura na universidade, uma vez que professores e outros trabalhadores acompanham os resultados (disponibilizados anonimamente). O *feedback* envolve o gerenciamento de tempo, melhores habilidades de estudo e metacognitivas, bem como preparação para as dificuldades específicas dos programas^44^. A UFSCar desenvolve pesquisa longitudinal de seguimento da comunidade universitária e criou programa de extensão envolvendo os departamentos acadêmicos de cursos da área da saúde, o Hospital Universitário, entre outras entidades, tendo como eixo o diagnóstico institucional, o perfil de saúde mental na comunidade universitária e a articulação com a rede de atenção psicossocial do município^29^.

**Mensagem-chave 11.** Baseou-se na experiência com o desenvolvimento de programas de tutorias em duas unidades da Universidade de São Paulo (USP): a FOUSP, que disponibiliza recursos como *site,* cartilha do tutor com orientações básicas para docentes, treinamento anual dos tutores com profissionais de saúde mental e relatórios semestrais de acompanhamento, com 20 docentes, uma psicóloga e duas assistentes sociais apoiando alunos de graduação^73^; a Escola de Enfermagem, que desenvolve projeto de tutoria acadêmica, com quatro docentes da Comissão do Curso de Bacharelado, sendo uma de referência para cada ano da graduação, para acompanhamento de dificuldades acadêmicas, escuta de sofrimentos e desgastes relacionados à saúde mental e encaminhamentos^74^. Trabalhadores da moradia estudantil da Universidade McMaster e membros das associações estudantis colaboram com os alunos do primeiro ano de graduação que apresentam dificuldades na transição para a universidade; além disso, os docentes são encorajados a oferecer aos estudantes, nas disciplinas, disponibilidade para discutir problemas de saúde mental, como estresse e ansiedade; outros grupos também oferecem apoio aos estudantes^33^.

## DISCUSSÃO

As opções formuladas a partir da literatura para responder às necessidades em saúde mental de universitários são desafiadoras e demandam que as universidades estabeleçam comissões institucionais capazes de implementar uma política na área.

A primeira opção convoca gestores de cursos da área da saúde e universidades à ação e à tomada de decisão, mostrando a necessidade do estabelecimento de políticas institucionais, em contraponto a ações pontuais e iniciativas isoladas. Essa decisão exige a constituição de um grupo de trabalho que: elabore um plano para implementação, com finalidades e metas claras e objetivamente delineadas; proporcione o desenvolvimento e monitoramento dos resultados dos projetos e ações implementadas; e ofereça suporte permanente a estudantes e dirigentes, para a efetivação e continuidade do processo. Essas ações não devem perder de vista o enfrentamento do estigma intrínseco ao tema do sofrimento mental, nem a perspectiva de tratar o tema da saúde mental transversalmente na matriz curricular, a fim de viabilizar ao estudante o desenvolvimento de análise crítica e a compreensão das raízes dos problemas enfrentados. Os estudos apontam a necessidade de inclusão de docentes e demais trabalhadores no processo de discussões, aperfeiçoando o debate e favorecendo propostas de enfrentamento conjunto, com compromisso institucional quanto a temas sensíveis que geram sofrimento, como racismo, preconceitos contra LGBTI e cotistas, e outras formas de discriminação^48,75-77^. Essa primeira opção exige das instituições profundas mudanças estruturais, de paradigmas e de práticas, e sua implementação no médio/longo prazo demanda dedicação de tempo e esforço dos envolvidos.

Como segunda opção, no curto prazo, as universidades e cursos da área da saúde podem esforçar-se para identificar ações existentes e serviços disponíveis, a fim de os integrar e ofertar formalmente à comunidade universitária informação e acesso a essa rede de cuidado. É imprescindível o protagonismo institucional, organizando programas e ações articulados à rede interna de cuidados da universidade e ao SUS, em detrimento de ações isoladas. Inclui-se a necessidade de ampliar a oferta desses serviços, apontando-se dois caminhos: ampliação das atividades terapêuticas para que não se restrinjam a intervenções farmacológicas, e inclusão de tecnologias de informação e comunicação para avaliar os sofrimentos, orientando e encaminhando os estudantes a serviços apropriados. A telessaúde é uma ferramenta útil para aumentar o acesso, sendo a saúde mental pioneira no uso dessas tecnologias^67,71,72^. A universidade, que muitas vezes pesquisa essas inovações, tem o dever de incorporá-las à rotina do cuidado de sua comunidade^16,17^.

A opção 3 enfoca a necessidade de desenvolvimento institucional de estratégias educativas que iluminem os problemas psíquicos contemporâneos, com destaque para aqueles advindos de preconceitos e atitudes de discriminação, e as formas de enfrentamento deles. A implementação de estratégias educativas e de debates com toda a comunidade acadêmica deve centralizar-se nas questões relacionadas às formas de discriminação e sua relação com o sofrimento psíquico. Esse debate pode trazer à tona demandas e proporcionar o fortalecimento da rede de apoio intra-institucional^31,33^.

A opção 4 evidencia que o enfrentamento do sofrimento psíquico requer o monitoramento permanente das ações em saúde mental, bem como sua inclusão na agenda institucional, com a criação de redes de apoio a estudantes e com o envolvimento de toda a comunidade acadêmica. Nessa opção, identificam-se programas de tutoria e monitoramento de casos de sofrimento mental entre os estudantes, no âmbito das unidades, que demandam estratégias de enfrentamento e acolhimento, reflexão conjunta dos problemas identificados e dos possíveis encaminhamentos, acompanhamento e monitoramento dos processos^28,43,73^. A instituição pode criar mecanismos de trabalho integrado com serviços de saúde, família e professores, para facilitar o processo terapêutico do estudante, dado que o meio universitário é excelente para a implementação e acompanhamento de ações de promoção da saúde mental^4,19^.

As necessidades em saúde mental são conformadas pelas formas de trabalho e vida inerentes à inserção de classe dos estudantes e suas famílias^78^. Uma política universitária de fortalecimento dos universitários da área da saúde, que tome em consideração as opções estratégicas evidenciadas na literatura, deve reconhecer as diferenças sociais e as diferentes manifestações de sofrimento psíquico dos estudantes, na implementação de mecanismos de monitoramento coletivo.

Em cursos da saúde, as condições de ingresso e permanência, bem como a ocorrência de problemas em saúde mental, são desiguais. Analisando a ocorrência de sintomas depressivos entre acadêmicos de diferentes cursos, identifica-se maior prevalência desses sintomas nos cursos de enfermagem, seguidos pelos cursos de odontologia e de medicina^79^. Esses cursos são frequentados por graduandos de grupos heterogêneos do ponto de vista da inserção de classe, considerando indicadores sociais como escolaridade do pai e da mãe e tipo de instituição (público/privado) em que o estudante cursou o ensino médio^80^.

Reconhecer que o ambiente universitário pode ser, em parte, causador do sofrimento mental é etapa fundamental na transformação da universidade em um ambiente mais saudável.

Para fazer enfrentamento de sofrimentos psíquicos, a UFSCar constituiu uma comissão que propôs uma política de saúde mental^b^ que prevê a integração entre serviços do SUS e os serviços e recursos oferecidos pela universidade, para desenvolver ações voltadas: ao aprimoramento da saúde mental; à prevenção de agravos; à prestação de cuidados a consequências desses possíveis agravos, como tentativas de suicídio; e à redução de danos ocasionados por uso problemático de substâncias psicoativas. Prevê também ações para coleta, análise e gestão de dados para gerar indicadores e monitoramento dos fenômenos do sofrimento psíquico e avaliar as ações desenvolvidas. Indica também o estabelecimento de mecanismos para compreender a relação entre os processos de ensino/aprendizagem e o sofrimento psíquico, bem como a elaboração de Código de Ética da UFSCar, e de protocolos para ações preventivas e para o cuidado a situações de violência^81^.

As opções aqui apresentadas, para o enfrentamento do sofrimento psíquico de estudantes, exigem envolvimento de toda a comunidade acadêmica, com marcante compromisso do corpo docente, que, contudo, encontra-se sob intensa pressão e carga psíquica^82^. No contexto brasileiro, aos docentes vinculados a programas de pós-graduação, somam-se cobranças para a produtividade científica, que geram sofrimento e adoecimento^83^.

A precariedade subjetiva sentida pelo professor universitário, permanentemente preocupado em responder às altas exigências de produtividade, traduz-se em sentimento de isolamento e abandono^84^. Essa precariedade opera de modo menos objetivo, se comparada à precariedade que acomete os trabalhadores de empresas terceirizadas e os professores temporários, pois estes estão submetidos ao trabalho informal e à perda de direitos fundamentais, sociais, trabalhistas e previdenciários. Por isso, veem sua segurança concretamente abalada.

Pós-graduandos também têm sido objeto de estudos relacionados a desgastes em saúde mental^9,85,86^, como consequência de pressões. Há pressões causadas pela obrigação de cumprir demandas acadêmicas; pela dificuldade em manter equilíbrio entre a vida acadêmica e pessoal; pelas incertezas com o futuro^85^; e pela necessidade de atingir as metas de produtividade acadêmica exigidas pelas agências de fomento^86^.

Portanto, a implementação de respostas às necessidades em saúde mental no âmbito das universidades encontra o contexto de pressão para o produtivismo, que impõe a docentes, estudantes e todo o conjunto de trabalhadores, o alcance de metas institucionais baseadas em padrões internacionais quantitativos de excelência acadêmica. O aprofundamento e amplificação do neoliberalismo na educação superior precisa ser explicitada e reconhecida como um processo que responde à agressiva expansão capitalista global, moldado pela lógica de mercado^87,88^. No caso brasileiro, questiona-se a qualidade da produção do conhecimento frente à atual competição para a excelência^89^.

É urgente que as universidades assumam compromissos com a causa pública, genuinamente democrática, para e com a sociedade. Sendo o neoliberalismo ideologicamente pedagógico, no sentido de ensinar a consentir e a reproduzir a dominação, a resposta, também de natureza pedagógica, deve se contrapor a ele radicalmente, constituindo-se como pedagogia transformadora, cuja finalidade é a liberdade e a emancipação^90^.

Este estudo apresenta algumas limitações: não levantou evidências em redução de danos, abordagem cada vez mais adotada pelas instituições, na área do consumo prejudicial de drogas entre estudantes universitários; algumas revisões não sistemáticas e estudos primários foram incluídos, em função da necessidade de abordar as dimensões coletivas e organizacionais e outros objetos relacionados que não foram abordados nas revisões sistemáticas.
